# Exploring Nurses’ Perceptions on Clinical Governance at Grey Hospital, Eastern Cape Province, South Africa

**DOI:** 10.3390/ijerph23060704

**Published:** 2026-05-26

**Authors:** Phiwokuhle Dike, Onke Ronaldy Mnyaka

**Affiliations:** 1 Health Policy and Global Health Division, School of Public Health, Walter Sisulu University, Mthatha 5099, South Africa; 230272002@mywsu.ac.za; 2 WSU Global Centre for Human Resources for Health Intelligence, Walter Sisulu University, East London 5247, South Africa; 3 WSU Centre for Clinical Governance and Healthcare Administration, Walter Sisulu University, East London 5247, South Africa

**Keywords:** clinical governance, quality improvement, nurses’ perceptions, district hospital, South Africa

## Abstract

**Highlights:**

**Public health relevance—How does this work relate to a public health issue?**

- Clinical governance plays an important role in improving quality, safety, and accountability in public health systems. Yet, there is limited information about how it is put into practice in under-resourced district and tertiary hospitals in low- and middle-income countries.
- This study assesses how nurses perceive the implementation of clinical governance, which managers are involved in, and the importance of their roles at Grey Hospital, a public district hospital in the Eastern Cape, South Africa.

**Public health significance—Why is this work of significance to public health?**

- The findings show that most nursing staff agree on the importance of managerial roles in clinical governance. This evidence can help guide the creation of clearer roles and accountability systems in public hospitals.
- Nurses are key frontline health workers who help put clinical governance into practice. Examining their views on management involvement can inform better evidence-based ways to improve governance in health systems with limited resources.

**Public health implications—What are the key implications or messages for practitioners, policy makers and/or researchers in public health?**

- Hospital managers and clinical governance staff should build on nurses’ shared views about the importance of management roles. By doing so, they can introduce participatory governance models that help improve accountability and service quality in their facilities.
- Policy makers and researchers should focus on developing clinical governance frameworks tailored to the needs of public hospitals in the Eastern Cape and similar areas. They should also support long-term studies to see how views on management involvement affect patient safety and health results.

**Abstract:**

Clinical governance is a systematic approach to maintaining and improving the quality of patient care within health systems. This research assessed nurses’ perceptions of the availability of clinical governance protocols and quality improvement activities at Grey Hospital, a district hospital in the Eastern Cape Province of South Africa. A descriptive cross-sectional study was used to collect quantitative data from doctors and nurses across various disciplines using a structured questionnaire. Data were captured using Microsoft Excel and analysed using STATA. Of the 105 participants who consented to participate, the majority were female (66.4%) and aged between 37 and 47 years (48.6%), with a mean age of 43.9 years. Most participants were professional nurses (59.8%), with enrolled nurses (20.6%) and enrolled nursing assistants (17.8%) comprising the remainder of the sample. The findings show that nurses perceived most clinical governance protocols to be present at Grey Hospital. However, only 54.3% reported occurrence of regular morbidity and mortality (M&M) meetings. These results suggest that while nurses perceive clinical governance protocols to be available at Grey Hospital, the gap in regular M&M meetings indicates that perceived availability does not automatically translate to effective implementation. Strengthening accountability mechanisms, ensuring adequate resources, and establishing systematic monitoring are essential to translate protocols into sustained quality improvement.

## 1. Introduction

Clinical governance is a system through which health service organisations are responsible and accountable for continuously working to improve the quality of their services, safeguarding high standards of care, and ensuring the best clinical outcome for patients [1,2]. The concept of clinical governance was first introduced in the United Kingdom in the late 1990s to tackle wide differences in quality of care throughout Britain [3]. Its purpose was to provide a framework supporting local National Health Service (NHS) organisations in implementing their statutory duties of quality. Clinical governance ensures the quality and safety of our public healthcare systems [4] by creating an environment conducive to excellence in clinical care [1]. It holds clinicians and healthcare organisations to a set of standards important for health and national health strategies [5].

Clinical governance encompasses seven interconnected pillars: (1) clinical effectiveness and evidence-based practice, (2) risk management and patient safety, (3) patient and public involvement, (4) education and training, (5) clinical audit and quality improvement, (6) information management, and (7) staff management and professional development. These pillars provide a comprehensive framework for systematically improving healthcare quality.

Clinical governance provides an opportunity for service providers to better understand and develop the fundamental components needed to deliver positive patient experiences and outcomes for their health issues [6]. Clinical governance also promotes a learning environment, one where healthcare professionals are encouraged to constantly build on their skills and improve their decision making and healthcare outcomes [5].

Despite a clear framework, a substantial gap persists between policy and practice, especially in under-resourced settings. Although clinical governance protocols may be formally established, they often do not lead to improved care quality when resources are limited, leadership is ineffective, or staff engagement is lacking. Assessing healthcare workers’ perceptions of protocol availability is therefore essential for identifying implementation gaps.

South Africa followed suit by adopting and introducing clinical governance into the country’s health system [7]. In 2018, the Minister of Health published a regulation entitled ‘Norms and standards applicable to different categories of health establishments to promote and protect the health and safety of users and healthcare personnel’ [8]. This regulation contains sub-regulations across the following domains: user rights; clinical governance and clinical care; clinical support services; facilities and infrastructure; governance and human resources; and general provisions [8]. This publication of regulations demanded a major shift in values, culture, and leadership to place a greater focus on the quality of clinical care and to make it easier to bring about improvement and change in clinical practice [9].

Nevertheless, South Africa continues to grapple with disparities in healthcare access and quality between rural and urban areas which significantly affect the implementation of clinical governance. Disparities create challenges that hinder the effective establishment and functioning of clinical governance through limited resources, variation in standard of care and challenges in data collection and monitoring [9]. Health professionals are crucial to the success of clinical governance, and their leadership and involvement in quality improvement, collaboration, education and monitoring efforts can help overcome these challenges and foster a culture of safety and quality in healthcare delivery [6]. In South Africa, there is a concentration of quaternary, tertiary, and regional hospitals in urban areas, leaving rural populations to rely on smaller district hospitals for health services [10,11]. Also, a minority of health professionals are servicing the rural district hospitals [9]. Reports by the Department of Health (DoH) indicate that urban areas have a higher density of doctors and nurses [10]. For instance, while the national average is about 0.8 doctors per 1000 people, rural areas have as low as 0.1 doctors per 1000 people resulting in limited access to medical care for rural populations [12].

District hospitals play a crucial role in the healthcare system, serving as primary points of access for a significant portion of the population with unique healthcare needs [6]. However, the unique contextual factors, resource constraints, and diverse healthcare needs prevalent in district settings present distinct challenges (or barriers) and can hinder the successful implementation of clinical governance. For example, government policies, societal norms, and cultural beliefs can either facilitate or hinder the implementation process [13].

The health system in the Eastern Cape is characterised by underdeveloped hospital services, shortage of health professionals and beds, inadequate access to quality specialist hospital services and generally poor health outcomes [7]. A report by the DoH linked some of these challenges to poor leadership and governance in healthcare facilities [7]. A well-resourced healthcare institution is better equipped to promote high standards of care, ensure patient safety and foster a culture of continuous improvement, leading to enhanced healthcare quality and outcome; thus, the availability of resources, both financial and human, within healthcare institutions significantly affects clinical governance implementation [5].

According to a report by the DoH, Grey Hospital is one of the hospitals which is performing poorly in the Buffalo City Metro Municipality, Eastern Cape Province [7]. The report noted with concern the weaknesses in the areas of clinical governance and management. These weaknesses in areas of hospital governance, including clinical governance, can have a negative consequence on the quality of healthcare services and healthcare outcomes of the population [11].

Grey Hospital was purposively selected for theoretical and practical reasons: (1) it is a representative district hospital: as a 67-bed district facility serving a rural/peri-urban population, Grey represents the hospital type where most South Africans access healthcare yet which receives less research attention than tertiary centres; (2) there are documented governance challenges: DoH reports specifically identified Grey Hospital as performing poorly on governance indicators, making it a critical case for understanding implementation barriers; (3) feasibility and access: institutional relationships facilitated data collection, enabling in-depth assessment; and (4) generalisability considerations: findings from a challenged district hospital provide insights which are potentially transferable to similar under-resourced settings. This case study approach allows detailed contextual examination to inform broader district hospital quality improvement strategies.

Existing clinical governance research predominantly focuses on tertiary hospitals in high-income countries, with limited evidence from African district hospitals or frontline healthcare worker perspectives. Specifically, no studies have examined nurses’ perceptions of clinical governance protocol availability in South African district hospitals, despite nurses constituting most of the healthcare workforce and being central to quality improvement implementation. This study addresses three gaps in the literature: (1) the frontline perspectives of most clinical governance research examine management and administrative views rather than frontline workers who operationalise governance; (2) a focus on district hospitals fills the evidence gap on clinical governance in resource-constrained, rural-serving facilities; (3) the perception–implementation divide explicitly distinguishes between perceived protocol availability and actual implementation, a critical conceptual distinction often conflated in the governance literature. Findings inform context-appropriate governance-strengthening strategies for similar settings.

Despite these findings, the extent to which clinical governance protocols and/or quality improvement initiatives are implemented at Grey Hospital remains unknown. No research study has been conducted to explore or establish the implementation of clinical governance at Grey Hospital. Thus, this study quantitatively assessed nurses’ perceptions of: (1) the availability of clinical governance protocols and quality improvement activities aligned with South Africa’s National Core Standards; (2) the perceived involvement of key hospital managers in clinical governance implementation; and (3) the perceived importance of these managers in sustaining quality improvement efforts at Grey Hospital.

### 1.1. Significance of the Study

This study provides insights on the availability and perceived implementation of clinical governance protocols or quality improvement activities at Grey Hospital. This study also provided information about the key players in the implementation of clinical governance at Grey Hospital as observed by doctors and nurses. Findings from this study provide baseline information, activate in-depth analysis on the implementation, or lack thereof, of clinical governance at Grey Hospital. Furthermore, the study makes recommendations on the important role of members of staff in ensuring that the clinical governance framework is implemented purposefully in a hospital.

### 1.2. Research Objectives

❖ To identify available clinical governance protocols and related quality improvement activities at Grey Hospital.
❖ To identify the principal role players involved in the implementation of clinical governance activities at Grey Hospital.
❖ To estimate the perceived level of importance of selected managers at Grey Hospital.

## 2. Materials and Methods

### 2.1. Study Design

We used a quantitative approach with a cross-sectional design to answer the research questions. The use of a quantitative research approach ensured that data were collected in a consistent manner, while the cross-sectional design allowed the researchers to collect data at a single point in time [14].

### 2.2. Study Setting

The study was conducted at Grey Hospital, a district-level hospital located in King William’s Town (Qonce) within the Buffalo City Metropolitan Municipality of the Eastern Cape Province, South Africa [15]. The catchment population of Grey Hospital is around 219,000 individuals, resulting in a bed-to-population ratio of 0.31 beds per 1000 inhabitants which is lower than the global and African regional averages of 2.7 beds per 1000 population and 1.0 beds per 1000 population, respectively [16]. Buffalo City is a metropolitan municipality situated along the east coast of the Eastern Cape, a province that spans approximately 168,966 square kilometres, accounting for 13.8% of South Africa’s total land area [15].

Grey Hospital comprises several departments, including Emergency, Paediatrics, Maternity, Outpatients, Surgical, Medical, Operating Theatre, Pharmacy, Counselling, Laundry, Kitchen, and Mortuary services. As a cornerstone of healthcare delivery in the region, Grey Hospital plays a critical role in providing essential medical services to a predominantly rural and peri-urban catchment population [15]. Despite its significance, the hospital faces persistent challenges such as staff shortages, limited resources, high patient volumes, and governance constraints that are directly relevant to the implementation and sustainability of clinical governance practices [15].

### 2.3. Population and Sampling

A census approach was employed, targeting all nurses who met the inclusion criteria (*n* = 107 eligible). This approach was chosen due to the relatively small nursing workforce at this district hospital, enabling a comprehensive assessment of perceptions across all departments. All 105 nurses constituted the sample for this study, yielding a response rate of 98.1%. Two nurses declined to participate or were unavailable during the data collection period. Although 8 doctors were also employed at Grey Hospital, only two (2) consented to participate, an extremely small sample that would have rendered any subgroup comparison statistically unreliable (see exclusion criteria below). The study therefore focuses exclusively on nursing staff.

### 2.4. Inclusion and Exclusion Criteria

❖ Only nurses who had worked at Grey Hospital for at least six months were considered for participation. This period was considered to be long enough for participants to have adequate knowledge about the hospital operations, including attendance at quarterly meetings, familiarity with clinical protocols, and observation of managerial involvement in governance activities. This period exceeds the typical three-month probation period for permanent staff and allows locum staff sufficient orientation time.
❖ All categories of nursing staff were eligible (permanent, contract, locum, and part-time) provided they met the six-month tenure requirement and worked at least 20 h per week at Grey Hospital, ensuring adequate institutional exposure. Nurses who were on short-term leave (2 weeks or less) remained eligible for participation.
❖ Only participants who were at least 18 years old of age were considered for participation.
❖ Participants who chose not to sign the informed consent form were excluded from participation. This criterion ensured that all included individuals voluntarily agreed to participate and understood the nature of the research.

### 2.5. Data Collection and Management

The researcher utilised a validated, structured quantitative questionnaire derived from Chitha’s “Clinical Governance Implementation Status Survey” (CGIS) [17]. The original CGIS contains 68 items across multiple domains. We selected 32 items specifically relevant to district hospital settings based on: (a) alignment with South African National Core Standards; (b) feasibility of assessment by frontline nursing staff; and (c) relevance to Grey Hospital’s operational context. Selected items covered: quality improvement meetings, infection control protocols, patient safety mechanisms, complaint management, clinical audit activities, and leadership involvement.

Content validity was assessed through expert review by two clinical governance specialists who confirmed that selected items adequately represented key clinical governance domains relevant to the study setting. The adapted questionnaire was piloted with 12 nurses at a similar district hospital to assess clarity, comprehension time, and face validity. Minor wording adjustments were made based on pilot feedback (e.g., changing “clinical governance committee” to “quality improvement committee” to reflect local terminology). Internal consistency was assessed using Cronbach’s alpha for the 32-item adapted scale: α = 0.84, indicating good reliability. For the 13-item managerial involvement subscale, α = 0.89.

Given that all prospective participants are professional healthcare workers, the questionnaire was administered in English without translation. Participants completed the printed questionnaires independently, although the researcher was available to provide clarification as needed. This approach helped mitigate social desirability bias by ensuring anonymity, minimising the data collector’s influence, and fostering a comfortable environment for respondents. As a result, this method facilitated the collection of accurate and genuine data, thereby enhancing the validity of the research findings.

The adapted CGIS questionnaire is structured as follows:

❖ Section A: Demographic profile. This section captured information about the nationality, sex, age, years of service, years of service at Grey Hospital and organisational status of the participants.
❖ Section B: Confirmation of the presence of the following quality improvement activities and protocols at the hospital. Participant responses used the following Likert scale options: 1 = Yes, 2 = No, 3 = Not sure, and 4 = Don’t know.
❖ Section C: Hospital staff members’ involvement in the implementation of quality improvement activities and their perceived level of importance. “Perceived level of importance” is defined as the degree to which nurses consider specific hospital managers as essential contributors to clinical governance implementation. The perceived importance scale used a 3-point Likert scale: 1 = Very Important, 2 = Somewhat Important, and 3 = Not Important.

All hard copies of data are stored in a secured, lockable cabinet accessible only to the researchers. Electronic data are stored in a cloud-based, password-protected system.

### 2.6. Data Analysis

Survey data were captured into Microsoft Excel version 2019 then exported into Stata version 18 for analysis. All analyses are descriptive: categorical variables are presented as frequencies and percentages, while continuous variables are reported as mean and standard deviation (SD) or median and interquartile range (IQR) depending on normality, assessed via the Shapiro–Wilk test. No inferential statistics were conducted. This approach is appropriate given the study’s descriptive objectives and census sampling design.

### 2.7. Ethical Consideration

Ethics approval was secured from the Human Research Ethics Committee of the Faculty of Health Sciences, Walter Sisulu University (Ref: 218/2024). Authorization to access the health facilities was granted by the Provincial Health Research Committee of the Eastern Cape (Ref: EC_202412_026). Furthermore, permission to conduct research at Grey Hospital was obtained from the hospital’s chief executive officer prior to data collection. The study adhered to the four ethical principles of autonomy, beneficence, non-maleficence, and justice. Participants were required to sign an informed consent form, ensuring that they were fully aware of their rights, including the right to withdraw from the study at any time or to decline to answer specific questions without facing any repercussions. All identifiable information, such as personal details, was removed from the data, ensuring complete anonymity.

## 3. Results

### 3.1. Demographic Characteristics of the Participants

Table 1 summarises the demographic characteristics of the study participants. Of the 105 participants who consented to participate 67.6% were female, of which the majority (47.6%) were aged between 37 and 47 years, with only 6.7% aged at least 59 years. The youngest participant was 26 years old, while the oldest was 60 years old with a mean age of 43,9 years (SD = 8.6). Most participants were professional nurses (59.8%), followed by enrolled nurses (20.6%) and enrolled nursing assistants (17.8%). In terms of years of service, 30.5% of the participants had at least 16 years of service, closely followed by those who ranged between having 11 and 15 years of service (28.6%), while those with between 6 and 10 and 1 and 5 years of service accounted for 21.0% and 20.0%, respectively. The years of service were relatively evenly distributed, with a median of 12 years (IQR = 10).

### 3.2. Availability of Clinical Governance Protocols and Quality Improvement Activities

**Figure 1:**
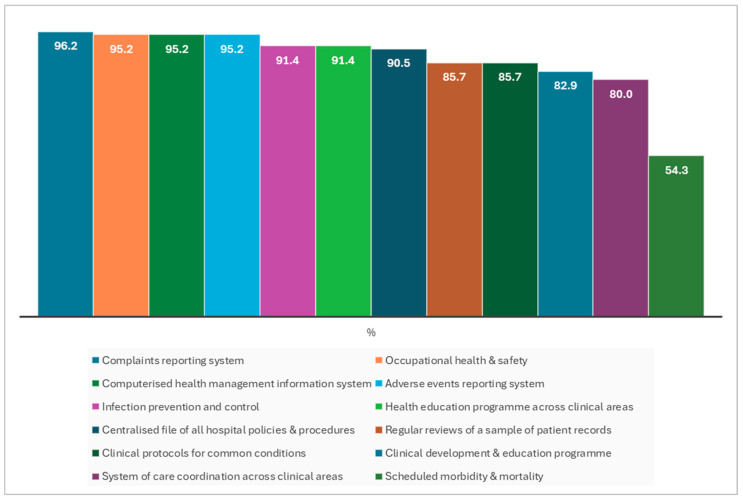
Distribution of participants’ responses on presence of clinical governance protocols and related quality improvement activities at Grey Hospital.

Figure 1 shows the percentage of nurses who confirmed (‘Yes’) concerning the presence of clinical governance protocols and related quality improvement activities at Grey Hospital. The results show that all clinical governance protocols and related quality improvement activities were affirmed present by at least 80% of the participants, except for scheduled morbidity and mortality meetings which was affirmed by 54.3%. Response options also included ‘No’, ‘Not sure’, and ‘Don’t know’. Across all items, ‘Not sure’ and ‘Don’t know’ responses ranged from 2 to 8%, suggesting most nurses felt confident in their knowledge of protocol availability.

### 3.3. Principal Role Players in Clinical Governance Implementation

Table 2 presents nursing staff perceptions of the principal players involved in the implementation of clinical governance protocols or activities, stratified by nursing category: PN (*n* = 64), EN (*n* = 22), and ENA (*n* = 19). Across all nursing categories and all thirteen management roles assessed, the predominant response was “fully involved”. Nursing-specific management positions attracted the highest levels of endorsement, with the nursing operations manager rated as fully involved by all PNs and ENAs (100% each), while all ENAs (100%) rated the nursing services manager as “fully involved” with near-unanimous ratings from PNs and ENs—98.4% and 95.9% respectively. Across all nursing categories, the clinical manager was rated as fully involved by a minimum of 89.1% of respondents, while the Head of Department was rated as fully involved by at least 92.2%. The quality assurance coordinator, infection control coordinator, and pharmacy supervisor each recorded “fully involved” responses exceeding 90.0% across all nursing categories. The CEO, corporate services manager, and information manager were similarly rated as fully involved by most respondents, with marginally greater variability noted across categories. The finance manager had the widest distribution of responses ranging from 79.7% of PNs, 90.9% of ENs, and 84.2% of ENAs selecting “fully involved”, with 12.5% of PNs selecting “sometimes involved”.

### 3.4. Perceived Importance of Selected Managers

Table 3 presents the nurses’ perceived level of importance of 13 managerial roles in clinical governance at Grey Hospital. The chief executive officer and finance manager were rated as “Very important” by all 105 respondents (100.0%). The corporate services manager received a “Very important” rating from 104 respondents (99.1%). The remaining ten roles comprising the clinical manager, head of clinical unit, nursing services manager, information officer, nursing operations manager, pharmacy supervisor, infection prevention and control manager, quality assurance manager, other health professionals, and X-ray department supervisor were each rated as “Very important” by 103 respondents (98.1%). Overall, the results show a high level of consensus among nursing staff regarding the importance of managerial roles in clinical governance at Grey Hospital.

## 4. Discussion

This study assessed nurses’ perceptions of the availability of clinical governance protocols or quality improvement activities, hospital management’s involvement and their level of importance in implementing clinical governance activities at Grey Hospital.

While nurses perceived a high availability of clinical governance protocols (>80% for most items), this contrasts with Grey Hospital’s documented governance challenges in DoH reports. This discrepancy suggests that protocol availability does not equate to effective implementation. Potential explanations include: (a) the protocols exist on paper but lack consistent application; (b) inadequate resources prevent operationalisation; (c) there is insufficient staff training on protocol use; or (d) there are weak accountability mechanisms for ensuring adherence.

The findings indicated that a significant number of clinical governance protocols and quality improvement activities were either available or actively taking place within the hospital. However, it was noted that fewer than 60% of participants reported the presence of scheduled morbidity and mortality meetings, indicating a potential gap in critical quality oversight mechanisms.

The finding that only 54% of nurses reported regular morbidity and mortality (M&M) meetings represents the most notable gap identified. Scheduled M&M meetings reflect an essential component of clinical governance, as they improve patient outcomes through reflective practice and learning from adverse events [18]. The low M&M meeting prevalence likely reflects multiple barriers prevalent in resource-constrained settings: inadequate staffing for meeting attendance, lack of protected time, limited senior clinical leadership, potential medico-legal concerns about documenting adverse events, and absence of standardised reporting systems. Research from similar South African district hospitals indicates that M&M meetings are often deprioritised due to operational pressures. 

While evidence from high-income settings demonstrates the effectiveness of M&M meeting, translating this to South African district hospitals requires addressing context-specific barriers. These include critical staff shortages preventing meeting attendance, lack of dedicated meeting time amid high patient loads, limited specialist oversight, and medico-legal concerns about documentation in under-resourced environments where system failures often underlie adverse events.

Findings regarding perceived managerial involvement may reflect different managers’ roles and visibility, for instance, clinical managers and nursing operations managers interact daily with frontline staff around quality issues, while corporate services or information managers work more behind the scenes on governance infrastructure (data systems, facilities) [19]. This differential visibility, rather than differential commitment, likely explains perception variations [19]. A similar study conducted at six NHS hospitals in England, the United Kingdom, [20] supports these findings.

These findings align with the National Core Standards for Health Establishments in South Africa, which explicitly require CEO accountability for quality and financial sustainability (Standards 1 and 5) [21,22]. The prominence nurses attribute to executive and financial leadership reflects an implicit understanding that clinical governance requires both strategic direction and resource commitment elements consistently identified in the implementation science literature as critical success factors. According to Lunn et al., CEO visible commitment correlates with successful clinical governance adoption [23]. While leadership commitment, supportive organisational culture, and access to reliable evidence collectively create conditions for strengthening governance structures and sustaining quality improvement efforts [24]. Moreover, the role of the finance manager is to enable governance through budgeting for quality initiatives, staffing investments, and infrastructure improvements [25].

The study indicates that participants regard the CEO and finance manager as important to the implementation of clinical governance at Grey Hospital. The high importance ratings above 90% for unit managers highlight the necessity for leadership at all levels to foster a culture of quality and safety. This reflects the study by [23] conducted in the United Kingdom which emphasises the CEO’s responsibility in creating a supportive environment for clinical governance. This involves allocating resources, setting strategic direction, and ensuring accountability for quality and safety [4]. Research suggests that a CEO’s visible commitment is directly correlated with the successful adoption of clinical governance frameworks. Furthermore, the finance manager’s role, often overlooked, is crucial in providing the necessary financial resources and incentives for clinical governance initiatives.

The high importance nurses attribute to executive leadership must be understood against the Eastern Cape health system’s documented governance challenges. Provincial audits reveal inadequate leadership capacity, financial mismanagement, and weak accountability at district level. That Grey Hospital nurses recognise CEO and financial leadership as important for clinical governance suggests awareness that institutional quality depends on addressing these systemic governance deficits protocol availability alone cannot compensate for leadership and resource gaps.

To enhance the framework, it is important to facilitate consistent information sharing among all staff members. Research demonstrates that organisational communication quality strongly predicts clinical governance implementation success: systematic information dissemination about governance activities, quality indicators, improvement initiatives, and outcomes fosters transparency, reinforces accountability, enhances staff engagement, and builds collective commitment to quality improvement [19]. Strategies could include regular meetings, workshops, and accessible digital platforms where staff can share insights, report concerns, and propose improvements. By prioritising continuous communication and the involvement of all personnel at Grey Hospital, the institution can strengthen its clinical governance framework, ultimately leading to improved patient outcomes and enhanced operational efficiency.

## 5. Limitations

This study has several limitations that warrant consideration. This study does not make any causal inferences due to its cross-sectional design. Reliance on self-reported nurse perceptions introduces potential bias and does not verify actual protocol implementation or effectiveness. The single-site focus restricts generalisability, while the exclusion of doctors means findings reflect only nursing perspectives. Measurement was confined to perceived protocol availability rather than adherence, quality, or patient outcomes, and the exclusion of “Not sure/Don’t know” responses may have inflated perceived availability. Collectively, these limitations indicate that the study provides a useful baseline snapshot but requires multi-site, longitudinal research with objective measures to comprehensively assess clinical governance in South African district hospitals.

## 6. Conclusions

The findings show that nurses at Grey Hospital believe most clinical governance protocols are available, with strong involvement from managers and a high value placed on executive leadership. Still, just because protocols are available does not mean they are always put into practice or followed effectively. There are some gaps, especially since M&M meetings are not held often, suggesting that formal protocols do not always lead to better quality practices. Nurses reported that most governance protocols were available (over 80%), except for M&M meetings (54%). They saw nursing operations managers, clinical HOD, and other health professionals as key stakeholders. Executive leadership, especially the CEO and the finance manager, was seen as most important, underscoring the importance of leadership and resource allocation for good governance.

To improve clinical governance, regular M&M meetings should be set up with dedicated time, clear guidelines, and consistent follow-up. There should also be objective audits to verify that protocols are being followed, along with feedback systems to keep frontline staff informed. Enough resources, like staff, training, and infrastructure, are needed to put protocols into practice. In addition, future research across multiple sites should compare staff perceptions with objective data, such as clinical audits, patient outcomes, and adverse event rates, to fully assess how well governance functions in South African district hospitals.

## Figures and Tables

**Table 1:** Summary of demographic characteristics.

Variables	N = 105	% = 100
Sex *n* (%)		
Male	34	31.7
Female	71	67.6
Age category *n* (%)		
26–36	20	19.0
37–47	50	47.6
48–58	28	26.7
≥59	7	6.7
Age, years; mean ± SD	43.9	8.6
	**Minimum**	**Maximum**
	26	63
Organisational status *n* (%)		
Professional nurse	64	61.0
Enrolled nurse	22	21.0
Enrolled nursing assistant	19	18.1
Years of service *n* (%)		
1–5	21	20.0
6–10	22	21.0
11–15	30	28.5
≥16	32	30.5
Years of service, median; IQR	12	10

Source: Survey data collected by the researchers (2025). Notes: SD = standard deviation; IQR = interquartile range.

**Table 2:** Participants’ responses on the principal players involved in the implementation of clinical governance activities.

Management Member	Nursing Category	Fully *n* (%)	Most Often *n* (%)	Sometimes *n* (%)	Rarely *n* (%)	Does Not *n* (%)	N
CEO	PN	57 (89.1)	6 (9.4)	1 (1.6)	0 (0.0)	0 (0.0)	64
	EN	21 (95.5)	0 (0.0)	1 (4.5)	0 (0.0)	0 (0.0)	22
	ENA	15 (79.0)	3 (15.7)	1 (5.3)	0 (0.0)	0 (0.0)	19
Corporate Services Manager	PN	55 (86.2)	9 (13.8)	0 (0.0)	0 (0.0)	0 (0.0)	64
	EN	19 (86.4)	2 (9.1)	1 (4.6)	0 (0.0)	0 (0.0)	22
	ENA	15 (78.9)	3 (15.8)	1 (4.8)	0 (0.0)	0 (0.0)	19
Finance Manager	PN	51 (79.7)	4 (6.3)	8 (12.5)	1 (1.6)	0 (0.0)	64
	EN	20 (90.9)	1 (4.6)	0 (0.0)	1 (4.6)	0 (0.0)	22
	ENA	16 (84.2)	1 (5.3)	2 (10.5)	0 (0.0)	0 (0.0)	19
Clinical Manager	PN	57 (89.1)	7 (10.9)	0 (0.0)	0 (0.0)	0 (0.0)	64
	EN	21 (95.5)	1 (4.6)	0 (0.0)	0 (0.0)	0 (0.0)	22
	ENA	17 (89.5)	1 (4.8)	1 (4.8)	0 (0.0)	0 (0.0)	19
Clinical Head of Department	PN	59 (92.2)	3 (4.7)	1 (1.6)	1 (1.6)	0 (0.0)	64
	EN	21 (95.5)	1 (4.6)	0 (0.0)	0 (0.0)	0 (0.0)	22
	ENA	18 (94.7)	1 (5.3)	0 (0.0)	0 (0.0)	0 (0.0)	19
Nursing Services Manager	PN	63 (98.4)	0 (0.0)	1 (1.6)	0 (0.0)	0 (0.0)	64
	EN	21 (95.9)	1 (4.6)	0 (0.0)	0 (0.0)	0 (0.0)	22
	ENA	19 (100.0)	0 (0.0)	0 (0.0)	0 (0.0)	0 (0.0)	19
Information Manager	PN	59 (92.3)	5 (7.7)	0 (0.0)	0 (0.0)	0 (0.0)	64
	EN	20 (90.9)	1 (4.6)	0 (0.0)	1 (4.6)	0 (0.0)	22
	ENA	18 (95.2)	1 (4.8)	0 (0.0)	0 (0.0)	0 (0.0)	19
Nursing Operations Manager	PN	64 (100.0)	0 (0.0)	0 (0.0)	0 (0.0)	0 (0.0)	64
	EN	20 (90.9)	1 (4.6)	1 (4.6)	0 (0.0)	0 (0.0)	22
	ENA	19 (100.0)	0 (0.0)	0 (0.0)	0 (0.0)	0 (0.0)	19
Pharmacy Supervisor	PN	58 (90.8)	5 (7.7)	1 (1.5)	0 (0.0)	0 (0.0)	64
	EN	20 (90.9)	1 (4.6)	1 (4.6)	0 (0.0)	0 (0.0)	22
	ENA	18 (95.2)	1 (4.5)	0 (0.0)	0 (0.0)	0 (0.0)	19
Infection Control Coordinator	PN	59 (92.2)	1 (1.6)	3 (4.7)	1 (1.6)	0 (0.0)	64
	EN	20 (90.9)	1 (4.6)	0 (0.0)	1 (4.6)	0 (0.0)	22
	ENA	17 (90.5)	0 (0.0)	1 (4.8)	1 (4.8)	0 (0.0)	19
Quality Assurance Coordinator	PN	60 (93.8)	1 (1.6)	2 (3.1)	1 (1.6)	0 (0.0)	64
	EN	20 (90.9)	1 (4.6)	0 (0.0)	0 (0.0)	1 (4.6)	22
	ENA	18 (95.2)	0 (0.0)	0 (0.0)	1 (4.8)	0 (0.0)	19
Other Health Professionals	PN	62 (96.9)	1 (1.6)	0 (0.0)	1 (1.6)	0 (0.0)	64
	EN	21 (95.5)	1 (4.6)	0 (0.0)	0 (0.0)	0 (0.0)	22
	ENA	18 (95.2)	0 (0.0)	0 (0.0)	1 (4.8)	0 (0.0)	19
X-ray Department Supervisor	PN	57 (89.2)	5 (7.7)	2 (3.1)	0 (0.0)	0 (0.0)	64
	EN	20 (90.9)	1 (4.6)	0 (0.0)	1 (4.6)	0 (0.0)	22
	ENA	18 (95.2)	0 (0.0)	1 (4.8)	0 (0.0)	0 (0.0)	19

PN = professional nurse; EN = enrolled nurse; ENA = enrolled nursing assistant.

**Table 3:** Perceived importance of selected managerial roles in clinical governance at Grey Hospital (*n* = 105).

Managerial Role, *n* (%)	N = 105	% = 100
Chief Executive Officer	105	100.0
Finance Manager	105	100.0
Corporate Services Manager	104	99.1
Clinical Manager	103	98.1
Head of Department—Clinical Unit	103	98.1
Nursing Services Manager	103	98.1
Information Officer	103	98.1
Nursing Operations Manager	103	98.1
Pharmacy Supervisor	103	98.1
Infection Prevention and Control Manager	103	98.1
Quality Assurance Manager	103	98.1
Other Health Professionals	103	98.1
X-ray Department Supervisor	103	98.1

Note. *n* = number of respondents rating the role as “Very important”. Two respondents did not rate selected roles, yielding a minimum valid *n* of 103 for 11 of the 13 roles.

## Data Availability

The raw data supporting the conclusions of this article will be made available by the authors upon request.

## Abbreviations

The following abbreviations are used in this manuscript:

NHS	National Health Services
DoH	Department of Health
CGIS	Clinical Governance Implementation Status Survey
CEO	Chief executive officer
BCMM	Buffalo City Metropolitan Municipality

## References

[1] Scally G., Donaldson L.J. (1998). Clinical governance and the drive for quality improvement in the new NHS in England. BMJ.

[2] Macfarlane A.J.R. (2019). What is clinical governance?. BJA Educ..

[3] Flynn M.A., Brennan N.M. (2020). Mapping clinical governance to practitioner roles and responsibilities. J. Health Organ. Manag..

[4] Thomas E.J. (2019). Improving teamwork in healthcare: Current approaches and the path forward. BMJ Qual. Saf..

[5] Balding C. (2019). From quality assurance to clinical governance. Aust. Health Rev..

[6] Walsh M.K. (2021). Clinical governance and patient experience. Glob. J. Public Health Med..

[7] Department of Health (2019). National Health Act (61/2003): Policy on National Health Insurance.

[8] Department of Health (South Africa) (2018). Norms and Standards Regulations Applicable to Different Categories of Health Establishments.

[9] Ghavamabad L.H., Vosoogh-Moghaddam A., Zaboli R., Aarabi M. (2021). Establishing clinical governance model in primary health care: A systematic review. J. Educ. Health Promot..

[10] Department of Health (South Africa) (2011). Human Resources for Health South Africa: HRH Strategy for the Health Sector 2012/13–2016/17.

[11] Coovadia H., Jewkes R., Barron P., Sanders D., McIntyre D. (2009). The health and health system of South Africa: Historical roots of current public health challenges. Lancet.

[12] Mburu G., George G. (2017). Determining the efficacy of national strategies aimed at addressing the challenges facing health personnel working in rural areas in KwaZulu-Natal, South Africa. Afr. J. Prim. Health Care Fam. Med..

[13] Whittaker S., Shaw C., Spieker N., Linegar A. (2011). Quality Standards for Healthcare Establishments in South Africa. S. Afr. Health Rev..

[14] Joubert G., Ehrlich R. (2012). Epidemiology: A Research Manual for South Africa.

[15] Eastern Cape Department of Health Grey Provincial Hospital. Eastern Cape Department of Health. https://www.echealth.gov.za.

[16] World Health Organization Regional Office for Africa (2022). Atlas of African Health Statistics 2022: Health Situation Analysis of the WHO African Region.

[17] Chitha W. (2015). The Implementation of Clinical Governance Protocols in the District Hospitals of or Tambo Health District, Eastern Cape Province, South Africa. Ph.D. Thesis.

[18] Pearson B. (2017). The clinical governance of multidisciplinary care. Int. J. Health Gov..

[19] Aveling E.L., Martin G., Armstrong N., Banerjee J., Dixon-Woods M. (2012). Quality improvement through clinical communities: Eight lessons for practice. J. Health Organ. Manag..

[20] Ferlie E., Fitzgerald L., Wood M., Hawkins C. (2005). The nonspread of innovation: The mediating role of professionals. Acad. Manag. J..

[21] National Department of Health, Republic of South Africa (2011). National Core Standards for Health Establishments in South Africa. https://www.health.gov.za.

[22] Gillam S., Siriwardena A.N. (2013). Frameworks for improvement: Clinical audit, the plan–do–study–act cycle and significant event audit. Qual. Prim. Care.

[23] Lunn M.L., Ellinger A.D., Nimon K.F., Halbesleben J.R. (2021). Chief Executive Officers’ Perceptions of Collective Organizational Engagement and Patient Experience in Acute Care Hospitals. J. Patient Exp..

[24] Mannion R., Davies H. (2018). Understanding organisational culture for healthcare quality improvement. BMJ (Clin. Res. Ed.).

[25] Ismail H.A., Kotp M.H., Basyouny H.A.A., Abd Elmoaty A.E.E., Hendy A., Ibrahim R.K., Abdelaliem S.M.F., Hendy A., Aly M.A. (2025). Empowering nurse leaders: Leveraging financial management practices to foster sustainable healthcare—A mixed-methods study. BMC Nurs..

